# Supplementary material for: GEM-based computational modeling for exploring metabolic interactions in a microbial community
Source: PLoS Comput Biol. 2024 Jun 20;20(6):e1012233. doi: 10.1371/journal.pcbi.1012233 (PMC11218945; doi:10.1371/journal.pcbi.1012233)
Supplement: Attachment — Submitted filename: respond-to-reviewer.docx [file pcbi.1012233.s001.docx]

Dear Editor

We appreciate your and the reviewers’ precious time spent reviewing our paper and providing valuable comments. It was your valuable and insightful comments that led to possible improvements in the current version. In total, we have added evaluations of well-studied pairwise interactions and communities of gut microbiome. Additionally, we renamed our methods and provided an extensive comparison with other methods in the Results and Discussion section.

We believe these revisions strengthen the article and address the concerns raised. The authors have carefully considered the comments and tried our best to address each one of them. We hope the manuscript, after careful revisions, meets your high standards. Please let us know if any further adjustments are needed. We appreciate your guidance and look forward to your final evaluation.

Thank you once again for your valuable input.

Sincerely,

Mojtaba Tefagh

On behalf of the authors

**Comments from Reviewer 1**

Soraya Mirzae et. al. have developed an algorithmic to predict the microbial interaction for co-culture systems. The authors have developed a graph-based algorithm, which does not require any biomass objective function and can predict microbial interaction under minimal growth conditions. The toy model of D. vulgaris and M. maripaludis has been used to validate the predictive ability of the algorithm by forming a pairwise community model. Possibilities of microbial competition, parasitism, commensalism, etc., have been explained by the pair of two species when they have shared common metabolites. Using the toy model, the authors have mentioned why metabolic uptake for species 2 is important to achieving the parasitic relation in association with species 1. To check the efficiency of the developed algorithm, they used the pairs of epiphyte strain Pantoea eucalypti (Pe229R) and different closely related phyllosphere bacterial strains. However, there are additional matters that require clarification in an updated version. Further detailed feedback is outlined below. However, there are few areas, which may be addressed by the authors for an overall improvement of their study.

1. Mixed-integer linear programming method has been used to simulate pairwise competition interactions for common metabolites. The problem statement not clear as the objective function for MILP is missing. The MILP code maybe provided for clarity.

**Response:** Thank you for pointing out the clarity issue. Most of the tools developed thus far for predicting the types of interaction involve an objective function, which can be either the sum of biomass reactions (used to compare growth rates) or the minimal set of metabolites in the medium. However, while the optimal value of the objective function is unique, the corresponding flux vector or medium may have an infinite number of possibilities [1]. Additionally, the definition of a community objective function is still an open problem [2].

Considering these issues, we consider a concept similar to MRO methods that account for common metabolites in the medium. Consequently, we view the problem from another perspective: metabolites that are essential for the survival of both species will be included in every medium. Therefore, the simultaneous absence of these metabolites would violate the survival of both species and other constraints we have imposed on the $F$ subspace**.** We impose the requirement of **non-zero exchange reactions (alternative medium) with constraint number 10 in formula**, along with the minimal growth rate **constraint** for both organisms, **numbered 5 and 6 in the formula**. By proposed definition, we can efficiently determine the competition for a common substrate in a metabolic network by solving the proposed MILP. Therefore, our approach does not need any prior knowledge of the community objective function.

We can consider the feasibility of the subset F, as a mixed-integer linear problem with an objective function of an arbitrary number, such as zero, and constraints defined in F. A feasibility problem aims to find a solution that satisfies a set of constraints without necessarily optimizing an objective function. In other words, does any flux v satisfy the constraints in F (can survive)? If the flux distribution $v$ involves non-zero exchange reactions and excludes substrate $i$, allowing both species to achieve a pre-specified growth rate, then the solver sets the objective function to 0. Otherwise, the problem remains infeasible. We also added more explanations into [Pg5-6, Ln159-203] [Availability of data and materials].

The code of MILP is available in: <https://github.com/mirzaei-s/microbial_interactions/blob/main/Competition.m> .

$\boldsymbol{maximize}$ **0**

$\boldsymbol{subject to:}$

$$\left[ \begin{matrix} v_{i}^{1}=0 \\ v_{i}^{2}=0 \\ \begin{matrix} v_{biomass}^{1}\geq\alpha{.v}_{biomass}^{1\_max} \\ v_{biomass}^{2}\geq\alpha{.v}_{biomass}^{2\_max} \\ \begin{matrix} S^{c}v=0 \\ lb \leq v \leq ub \\ \begin{matrix} \theta_{k}.\beta\leq v_{k} , \forall k \in Exchange \\ \sum_{k\in Exchange} \theta_{k} \geq1 \\ \theta_{k} \in\left\{ 0,1 \right\}, v \in R^{n} \geq0, 0<\alpha,\beta\leq1 \end{matrix} \end{matrix} \end{matrix} \end{matrix} \right]$$

2. Throughout the modeling work, authors have used α = 0.1 and β = 0.01 as the maximum biomass flux (for species 1) and minimum exchange flux (for species 2). It is very much unclear how these values were obtained.

**Response:** In general, the values of α and β depend on the specific application and can be chosen experimentally. However, in our problem, we select these values based on relevant articles, as this value is commonly used to impose a minimum level of growth in most of the methods proposed in [3]. [Pg7, Ln229-231].

3. From Fig 3, it has been seen that species 2 can survive in the absence of ammonia, as it has an alternative pathway to produce ammonia. Therefore, we can say that the developed method can also be helpful in predicting important pathways for coexisting species. In the era of genetic engineering, this might be a useful tool. Authors can think about that and elaborate their hypothesis to improve the usefulness of their tool.

**Response:** We appreciate the reviewer’s careful feedback. In genetic engineering of microorganisms with goal of achieving maximum efficiency, it is essential to understand inter-organism interactions and how they compete for resources. Identification and measurement of interactions within microbial community (MC), and accurate representation of these in theoretical models, is an important basis for building, engineering and control of MCs in the environment [2,4]. [Pg2, Ln56-58].

Our strategy assesses the natural inter-organism interactions. It predicts metabolites that are essential for the survival of species. Consequently, every metabolite that competes for its use can consider important pathways that interrupts the coexisting species [Pg5-6, Ln159-203]. As you suggested, we included this as future work in the conclusion. [Pg16, Ln629-631].

4. Is there any natural co-occurrence for the selected microbial species (toy models and test models)? If yes, please discuss a few sentences in the introduction part. So that readers can understand and correlate the modeling work with the naturally existing microbial community.

**Response:** We apologize for the lack of clarity on this matter. According to your suggestion, we revised this section. We hope that these revisions and the improved text will be satisfactory. [Pg3, Ln72-90].

5. How this method can be extended to community of three microbes or more.

**Response:** Thank you for bringing these issues to our attention. As discussed in the article, when the flux of one reaction increases for common metabolites, it depletes the substrate concentration, leading to a decrease in the flux of the opposing reaction. Considering this, two species compete for a common substrate$i$ if the logical expression $v_{i}^{2}=0\Longleftrightarrow v_{i}^{1}\neq0$ holds true. Using the same logical expression, for three species, we have:${(v}_{i}^{2}=0 {\wedge v}_{i}^{3}=0)\Longleftrightarrow v_{i}^{1}\neq0$

By using the logical equivalence:

$$\left[ v_{i}^{1}\neq0\Longrightarrow{(v}_{i}^{2}=0 {\wedge v}_{i}^{3}=0) \right]\wedge\left[ {(v}_{i}^{2}=0 {\wedge v}_{i}^{3}=0)\Longrightarrow v_{i}^{1}\neq0 \right]$$

$\equiv(v_{i}^{1}=0\vee{(v}_{i}^{2}=0\wedge v_{i}^{3}=0))$ $\wedge$ (${(v}_{i}^{2}\neq0 {\vee v}_{i}^{3}\neq0\vee v_{i}^{1}\neq0$)

$\equiv$($v_{i}^{1}=0 \vee v_{i}^{2}=0$) $\wedge(v_{i}^{1}=0 \vee v_{i}^{3}=0) \wedge$ (${(v}_{i}^{2}\neq0 {\vee v}_{i}^{3}\neq0\vee v_{i}^{1}\neq0$)	(1)

If the negation of Eq (1) holds true, then we would not have competition we have:

$\neg Eq\left( 1 \right)\Longrightarrow$ ($v_{i}^{1}\neq0 \wedge v_{i}^{2}\neq0$) $\vee$ $(v_{i}^{1}\neq0 \wedge v_{i}^{3}\neq0) \vee$ ${(v}_{i}^{2}=0 {\wedge v}_{i}^{3}=0\wedge v_{i}^{1}=0$)

For expression ($v_{i}^{1}\neq0 \wedge v_{i}^{2}\neq0$) and $(v_{i}^{1}\neq0 \wedge v_{i}^{3}\neq0)$, they imply that the concentrations of metabolite $i$in environment are sufficiently high. It is evident that when metabolite is abundantly available, species will not compete. So, if expression$(v_{i}^{1}\neq0\vee v_{i}^{2}\neq0\vee v_{i}^{3}\neq0)$ holds true (along with other constraints), it implies that **three species compete for its consumption. We can say that in order not to have competition, the** negation of the expression must hold true, which means ${(v}_{i}^{2}=0{\wedge v}_{i}^{2}=0 {\wedge v}_{i}^{3}=0)$ and that’s equivalent to the feasibility of subset $F$ with constraints ${(v}_{i}^{2}=0{\wedge v}_{i}^{2}=0 {\wedge v}_{i}^{3}=0).$We have added the suggested content to the manuscript on [Pg7, Ln247-268].

**Minor (reviewer 1)**

1. In line number 60, the authors have written that ‘we develop genome-scale computational modeling’. There is a lack of clarity in the statement, as they have utilized the published GEM models from Schlechter et al., 2023 (Ref. 36), which is mentioned in the “Materials and methods” section (Line no. 74).

**Response:** We are sorry for our careless mistakes in the manuscript. We mean stoichiometric-base modeling OR constraint-based modeling. We corrected this mistake to constraint-based modeling in the manuscript. [Pg3, Ln64-66].

2. Authors have mentioned that ‘All reactions in these models are reversible.’ while discussing the details of the ‘Toy model’. However, it has been observed that only the exchange reactions are reversible (according to Fig 3). Either change the line or discuss it properly.

**Response:** All the reactions are converted into two irreversible reactions. **In Fig 2, we have depicted these** irreversible **reactions with double lines. Additionally, we indicate irreversible reactions using arrows for the forward and backward reactions.** According to your suggestion and valuable comment, we reproduced the figure with high clarity to make this issue more distinguishable. [Pg4, Ln125-127]

3. Species names should be in italics. Throughout the manuscript, these have been written in normal text like ‘Desulfovibrio vulgaris’ (In line number 96).

**Response:** Thank you very much for your nice reminder. Revised accordingly.

4. A sentence should not be started with “we…” that is in line number 74. Instead of writing ‘ref.36’, please mention proper referencing of the article, from where the metabolic networks have been obtained.

**Response:** Thank you very much for your nice reminder. Revised accordingly.

5. In lines number 158 and 159, a fraction of the sentence (‘according to the definition’) appeared twice.

**Response:** Revised accordingly.

6. In Table 1, ‘compertition score’ might be ‘Competition score’. Please check.

**Response:** Thanks very much for your patient review. Revised accordingly.

7. The clarity of the provided figures should be improved.

**Response:** We apologize if our figures in our manuscript lacked sufficient clarity. We did not intend to attach images with low-quality, however, the clarity diminished upon uploading. We have modified the figures and hope they are now clear and concise. However, when uploading the figures, the contrast is reduced. In the right corner of the page, there is a link that you can click on to download the high-quality figures.

**Comments from Reviewer 2**

The interaction between microbial communities and their surrounding environment constitutes a subtle ecosystem. It is worth exploring how microorganisms interact, compete, and coexist with each other. This manuscript could be accepted if the following points are taken care of.

1. The linear programming algorithm established in microbial parasitism can consider mutually unfavorable influencing factors and the adverse environmental conditions faced together. If so, how is it reflected? If not, provide your reasons.

**Response:** Thank you for your question. Yes, our method can handle both mutually unfavorable and adverse environmental conditions. This is achieved not during the optimization problem itself, but rather in the preprocessing stage. We added the complete algorithm for this section in the Fig 3. As you can see, we first use FBA to obtain the growth rate of each species. Although we haven’t explicitly stated it at this stage, the assumption is that each species has an acceptable growth rate. If the environmental conditions are unfavorable for both species, the FBA results will yield low values, indicating that this environment is not suitable for either of them, and the researcher can disregard further algorithm steps.

In our parasitic/commensal examination, we restrict our analysis to a feasible space that ensures the survival of both organisms, by determining that each microorganism has the pre-defined growth rate. The giver species, at its maximal theoretical biomass rates and capable of producing the required compound, and the demanding species, which is in a survival condition, request from the opposite species its minimal needs ($v_{j}^{2(min-flux)})$, aiming to achieve its maximal theoretical biomass.

[Pg5-6, Ln156-200] [Pg8, Ln284-293]

2. The meanings of the numbers 19 and 23 in formula (16) need to be described in the paper.

**Response:** We apologize for the oversight. The numbers indicate the constraints’ numbers in the optimization problem. Formula (20) has been updated and we also added more explanations into [Pg10, Ln376-378].

3. The content in the image is very blurry, and you need to effectively improve the clarity of the image. A small number of annotations should be added next to the image to help readers understand.

**Response:** We apologize if our figures in the manuscript did not have sufficient clarity. We did not intend to attach images with low quality, but when we uploaded them, the clarity of the images reduced. We have modified the figures and hope that they are now clear and concise. However, when uploading the figures, the contrast is reduced. In the right corner of the page, there is a link that you can click on to download the high-quality figures.

**Comments from Reviewer 3**

The paper presents a GEM-based computational modeling approach for exploring metabolic interactions in a microbial community. The authors developed a computational model for a synthetic microbial community that can predict possible metabolite interactions between species. The approach was validated using a toy model and a syntrophic co-culture of Desulfovibrio vulgaris and Methanococcus maripaludis. The approach was also applied to a real-world case study involving Pantoea eucalypti 299R and six different phyllosphere bacteria. The following concerns should be addressed:

1. The authors should consider comparing your approach with several existing state-of-the-art methods for studying metabolic interactions in microbial communities.

**Response:** Thank you for the comment. As suggested by the reviewer, we have added a section to explain and compare existing methods in the discussion and results, and we have included this information in Table1. [Pg9-10, Ln312-355]

We compared with growth-based methods such as the OptCom and MICOM that similar to our method are static. We have used Interaction_Strength metrics described in [5] to predict the types of interaction as are positive or negative. [Pg12-13, Ln474-502].

OptCom and MICOM are two flux balance analysis frameworks for predicting the growth rate of each participant in the community. The results derived from these methods are included in Table 2. [Pg14, Ln514-523] [Pg14, Ln531-536]

2. There is a lack of comparisons with widely-known baselines in the field. More real-world datasets should be used to validate your methods along the comparing with current methods.

**Response:** Thank you for pointing out the clarity issue. We have included well-studied pairwise interactions and communities of gut microbiome in both the Materials and Methods section and the Results and Discussion section.

The existing algorithms have been evaluated on different and diverse datasets. There is a lack of methods that compare and evaluate themselves on common datasets with other methods. Considering the mentioned limitations in our manuscript, we tried using well-studied case, and explored their interactions empirically.

We have revised our manuscript according to your comments. We added two additional species, *Geobacter sulfurreducens* and *Rhodoferax ferrireducens*, which exhibit remarkable abilities to perform dissimilatory metal reduction in anoxic subsurface environments. [Pg3, Ln72-82], [Pg4, Ln136-142]

Previous studies have reported that these two species compete metabolites. [Pg11, Ln416-431]

Additionally, we also applied our framework to a honeybee gut microbiome community [Pg3, Ln83-90]. The honeybee gut microbiome exhibits numerous similarities with the human gut microbiome and is used as a model system in the article [6]. We have added the suggested content to the manuscript on [Pg12, Ln435-468]

3. The references introducing the backgrounds, particularly those pertaining to similar methods, appear to be somewhat outdated.

**Response:** Thank you for the comment. We found another similar method that was published recently in 2024. [Pg11, Ln416-431]

We have added a section to explain and compare existing methods in the discussion and results, and we have included this information in Table1. [Pg9-10, Ln312-355].

We hope that these revisions and the improved text will be satisfactory. [Pg12-13, Ln474-523] [Pg14, Ln534-539]

**Have the authors made all data and (if applicable) computational code underlying the findings in their manuscript fully available?**

**Response:** It is mentioned in the article [Pg3, Ln99, 101], [Pg4, Ln131] where all the data and code can be found, and we also added Availability of data and materials section.

[1] Smallbone, Kieran, and Evangelos Simeonidis. "Flux balance analysis: a geometric perspective." *Journal of theoretical biology* 258.2 (2009): 311-315.

[2] Perez-Garcia, Octavio, Gavin Lear, and Naresh Singhal. "Metabolic network modeling of microbial interactions in natural and engineered environmental systems." *Frontiers in microbiology* 7 (2016): 186528.

[3] Zomorrodi, Ali R., et al. "Mathematical optimization applications in metabolic networks." *Metabolic engineering* 14.6 (2012): 672-686.

[4] Widder, Stefanie, et al. "Challenges in microbial ecology: building predictive understanding of community function and dynamics." *The ISME journal* 10.11 (2016): 2557-2568.

[5] Joseph, Clémence, et al. "Predicting microbial interactions with approaches based on flux balance analysis: an evaluation." *BMC bioinformatics* 25.1 (2024): 36.

[6] Oftadeh, Omid, et al. "A constraint-based framework to reconstruct interaction networks in microbial communities." *bioRxiv* (2024): 2024-01.
